# SOCIAL CLASS AND SOCIAL TRUST: THE CASE OF A NORDIC WELFARE COUNTRY

**DOI:** 10.1093/geroni/igae098.3204

**Published:** 2024-12-31

**Authors:** Marja Jylha

**Affiliations:** Tampere University, Tampere, Pirkanmaa, Finland

## Abstract

Trust in societal institutions and other people is an important foundation of democratic systems and contributes to the quality-olf-life. Finland has been considered as a high-trust society, with a reasonably egalitarian society and educational system, and the welfare state, including universal public services. In recent years the availability of health care and home and long-term care for older people has decreased, and”care poverty” has increase. In the project”Aging and social well-being” we investigated the trust of older individuals in societal instutions and health and old people´s services, and the differences in trust between social classes, measured as education, income, and occupational social class. The nation-wide survey included 3085 invdividuals aged from 65 to 84 years. Of the five societal institutions incluced, schools and educational system was the most trusted, and business life the least trusted. Trust in each five societal institutions and for the summary score showed a linear association with each measure of social class. For health and care services, a clear majority in each social class trusted acute health care and care services for chronic conditions, but a clear minority trusted home care, and only one in fourth trusted long-term care. Trust in health care was associated with social class, but trust in older people´s services was low in all social classes. The findings show that even in a Nordic welfare state, trust in societal institutions is highly dependent on social position, but low trust in old people´s care is shared by all social classes.

